# Pre-Pregnancy Adherence to Mediterranean Diet and Risk of Gestational Diabetes Mellitus: A Prospective Cohort Study in Greece

**DOI:** 10.3390/nu15040848

**Published:** 2023-02-07

**Authors:** Antigoni Tranidou, Themistoklis Dagklis, Emmanuella Magriplis, Aikaterini Apostolopoulou, Ioannis Tsakiridis, Violeta Chroni, Eirini Tsekitsidi, Ioustini Kalaitzopoulou, Nikolaos Pazaras, Michail Chourdakis

**Affiliations:** 1 3rd Department of Obstetrics and Gynecology, School of Medicine, Faculty of Health Sciences, Aristotle University of Thessaloniki, 54642 Thessaloniki, Greece; 2 Department of Food Science and Human Nutrition, Agricultural University of Athens, 11855 Athens, Greece; 3 Laboratory of Hygiene, Social & Preventive Medicine and Medical Statistics, School of Medicine, Faculty of Health Sciences, Aristotle University of Thessaloniki, 54124 Thessaloniki, Greece

**Keywords:** Mediterranean diet, MD, Gestational Diabetes Mellitus, GDM, pregnancy, maternal nutrition

## Abstract

Gestational Diabetes Mellitus (GDM) is a growing epidemic affecting pregnant women and their offspring. This study aimed to identify the relationship between adherence to a Mediterranean diet (MD) before conception and the risk of GDM in a contemporary Greek pregnant cohort. A prospective cohort of pregnant women was recruited at the routine first trimester visit. Nutritional intake was evaluated using a population specific validated food frequency questionnaire (FFQ). Pre-pregnancy adherence to MD was derived using two different scoring systems, the Mediterranean diet index score (MDS), and a modified version. Adjusted odds ratios (aOR) were computed using multiple logistic regression models for each score derived. Of 743 participating women, 112 (15.1%) developed GDM. The MDS index showed that scoring 5–9 points (high adherence) was associated with a lower GDM incidence (aOR: 0.57 95% CI (0.32, 0.90), *p* = 0.02), while the modified MDS index showed no significant association for any level of adherence. Pre-pregnancy consumption of “meat and derivatives” and “fatty meat and processed meat” was associated with a higher risk of GDM, with both scoring systems (*p* = 0.008, *p* = 0.004, respectively). A higher adherence to a MD pre-pregnancy, especially with less meat consumption, may have a protective effect on the occurrence of GDM.

## 1. Introduction

GDM is a common carbohydrate intolerance affecting pregnant women worldwide, with different ethnic, behavioral, and cultural backgrounds [1,2]. Pregnancies with GDM are considered high-risk, as they are associated with a series of adverse outcomes, such as caesarean delivery, preeclampsia, macrosomia, preterm birth, and stillbirth [3]. Moreover, the pathophysiologic dysregulation that occurs in GDM may also have an impact in later life for both the mother and the offspring; it is a crucial determinant of healthcare cost and influences the quality of life of those affected [4,5,6,7,8]. Additionally, due to the absence of unanimous consensus among guidelines on the diagnosis and management of GDM, a number of cases may escape the appropriate attention. A recent comparative review by Tsakiridis et al. reported on the differences among the national and international guidelines regarding screening for GDM [9]. More specifically, guidelines by the International Federation of Gynecology and Obstetrics (FIGO,) the Australasian Diabetes in Pregnancy Society (ADIPS), the Society of Obstetricians and Gynecologists of Canada (SOGC), and the American College of Obstetricians and Gynecologists (ACOG) recommend screening for GDM at 24–28 weeks of gestation for all individuals, in the absence of other risk factors, whereas in the presence of additional risk factors, screening should be employed earlier. In contrast, the guideline by the National Institute for Health and Care Excellence (NICE) suggests screening at 24–28 weeks of gestation only for those that have risk factors. The Endocrine Society (ES) suggests universal screening at the first trimester for all individuals and, if negative, retesting at 24–28 weeks of gestation; the FIGO guidelines adopt this method for screening only in countries with increased risk for GDM occurrence. Finally, the American Diabetes Association (ADA) does not have specific recommendations on GDM screening. It should be noted that approximately 1–2% of all pregnancies are diagnosed with pre-gestational diabetes. Moreover, for women whose pregnancies were complicated with GDM, a glycemic test between six to twelve weeks following delivery is universally proposed.

Nutritional and lifestyle characteristics have been associated in variable degrees with proximate and/or long-term consequences for both the pregnant woman and the fetus [10]. Some studies have assessed the effect of maternal characteristics and nutritional aspects, such as the level of adherence to the Mediterranean diet (MD) during the pre-gestational or gestational period on the risk of developing GDM [11,12]. Olmedo-Requena et al. reported that high MD adherence prior to pregnancy was associated with lower incidence of GDM, whereas Assaf-Balut et al. reported that a MD pattern further supplemented with extra-virgin olive oil (EVOO) and pistachios during early pregnancy also reduced the risk of GDM. Additionally a study by Izadi et al. suggested that the higher the adherence to a lower Dietary Approaches to Stop Hypertension (DASH) or MD diet, the lower the rates of GDM are [13]. In addition, the preventive effect of physical activity both prior and also during pregnancy on lowering the incidence of GDM has been described [14,15,16]. Moreover, results of a recent umbrella review on the role of exercise in pregnancy indicated that the earlier the initiation of exercise was, the more favorable the prevention of GDM occurrence was [17]. Furthermore, the beneficial role of adherence to a MD diet in preventing or treating Type 2 diabetes mellitus (T2DM) in the general population has also been reported [18]. T2DM may have common mechanisms of pathogenesis with GDM; this has led to the hypothesis that MD may also act protectively against the pathogenesis of GDM. A number of studies conducted both in Mediterranean and non-Mediterranean populations have assessed adherence to MD or other maternal dietary patterns before pregnancy, but have reported on a variable degree of effect between MD adherence and the different dietary patterns used on the occurrence of GDM [19,20]. Study findings may vary due to the application of different tools for evaluating the adherence to MD in the non-Mediterranean populations, as their dietary habits differ from those of the Mediterranean populations [21,22,23].

Adherence to the original MD can be measured with a variety of tools, such as the Mediterranean Diet Score (MedDietScore) [24], the Mediterranean Diet Pyramid [25], the Mediterranean Diet Adherence Screener (MEDAS) [26], and many other tools developed around the world [27]. In Greece, the MDS that was developed by Trichopoulou et al. is widely used to assess adherence to the MD [28]. Modified versions have also been created to adapt to population specific dietary intakes and accommodate lifestyle changes while maintaining the primary MD categories, such as fruits and vegetables and monounsaturated fatty acids (MUFA) as the main fatty acid consumed. In the Greek field, Panagiotakos et al. also developed an index known as MedDietScore [24] based on the Mediterranean Diet Pyramid [25]. This scoring system includes 11 food groups and differs from the MDS as it includes potatoes and olive oil instead of the ratio used by the MDS for MUFA: SFA calculation, nuts are not included, while meat and poultry are considered negative factors. The effects of these diet scores on GDM risk have not to date been explored, in the same cohort, to identify potential MD score differences. 

Evidence suggests that composition of gut microbiota may play a role in the modulation of glucose metabolism and might be the intermediary between gut microbiome alterations and onset of GDM [29,30]. The exact interplay between these changes is not yet clearly identified, but it is suggested that changes in the composition of the gut microbiome, along with the occurrence of insulin resistance that develops during pregnancy, may have an impact in energy homeostasis affecting intestinal permeability [31]. Additionally, a recent systematic review on the relation of gut microbiome and GDM pathogenesis revealed differences in the gut microbiome during the first trimester of pregnancy in women with post-GDM diagnosis, but no specific contributor was identified [32]. Interestingly, a current study by Pinto et al. observed that the associated GDM-changes in the gut microbiome may have preceded the occurrence of GDM by more than ten weeks before the typical diagnosis of GDM [33].

Dietary habits affect the composition of gut microbiota and have a significant impact on how the brain and behavior are modulated [34]. The synergistic effect of nutrient status, gut microbiota, and host environment play a key role in the modulation of the gut–brain axis which is responsible for health and disease. Gut microbiome influences brain function by processing the nutrient intake and by synthesizing metabolites. Altering the nutrient intake through diet can have an impact on how the brain and behavior work. High adherence to the Mediterranean diet has a beneficial effect on the gut microbiome and has been associated with promoting health [35]. Exploring the dietary patterns of women before pregnancy may further elucidate on the relationship of dietary habits and GDM occurrence.

To our knowledge, no study in the Greek population has examined the relationship of maternal adherence to MD prior to conception and its effect on GDM. Thus, the aim of this study was to evaluate the level of adherence to the MD and to a modified MD, six months prior to conception, and the associated risk of GDM in a Greek pregnant cohort.

## 2. Materials and Methods

### 2.1. Study Design and Participants

This was a prospective study targeting pregnant women that attended the 3rd Obstetrics and Gynecology Department, Aristotle University of Thessaloniki, Greece. This study included participants from a large cohort study, named “BORN2020” that commenced in Thessaloniki in 2020, and is ongoing, with the aim to collect and analyze data among women before and during pregnancy. We evaluated their adherence to MD six months prior to pregnancy. All participants were recruited at their routine antenatal visit for their ultrasound check 11^+0^–13^+6^ weeks of gestation. Of note, the national antenatal protocol recommends a universal ultrasound check at 11^+0^–13^+6^ weeks of gestation. 

An approval by the Bioethics Committee of the Aristotle University of Thessaloniki was obtained (5/12.4.2022). Individuals were informed about the study and if they were positive, consent was obtained.

All pregnant women attending for their antenatal visit were eligible. Exclusion criteria were as follows: (i) age <18 years, (ii) serious pre-existing medical condition (e.g., chronic hypertension, pre-existing diabetes), (iii) women on diets that exclude specific dietary products due to medical conditions or lifestyle (e.g., vegetarian, vegan, gluten sensitivity, etc.), (iv) previous history of GDM, polycystic ovary syndrome or acanthosis nigricans, or women on corticosteroid medication.

The results were adjusted for known risk factors of GDM, including age > 35 years and overweight or obesity, to minimize the effects of these imbalances. In our dataset, although no exclusions were employed on nationalities, all candidates were of Greek origin.

For the diagnosis of GDM, all women underwent a 75 g oral glucose tolerance test (OGTT) at 24–28 weeks of gestation, following the criteria suggested by the Hellenic Society of Obstetricians and Gynecologists (HSOG) [36], which are based on the Hyperglycemia and Adverse Pregnancy Outcomes (HAPO) study [37]. Thus, the diagnosis of GDM was set when at least one of the measurements of blood glucose was equal or above the predefined thresholds: (i) fasting ≥92 mg/dL, (ii) 1-h ≥180 mg/dL, (iii) 2-h ≥153 mg/dL [38]. 

### 2.2. Variables for Assessment

Maternal anthropometric and habitual data were recorded from each participant at their visit at the antenatal clinic. Height was measured in centimeters (cm) using a stadiometer. Current weight was also measured in kilograms (kg), and pre-pregnancy weight was reported by the women. Based on BMI classification standards, women were categorized as underweight (BMI < 18.5 kg/m^2^), normal weight (BMI 18.5–24.9 kg/m^2^), overweight (BMI 25–29.9 kg/m^2^), or obese (BMI ≥ 30 kg/m^2^) [39]. Status of smoking before and during pregnancy was recorded and women were divided in past smokers, current smokers, or never smokers.

### 2.3. Assessment of Diet

Dietary assessment was performed using a locally validated FFQ, which was developed in order to evaluate the nutritional habits among pregnant women in a Greek population [40]. The FFQ was based on previous FFQs that have been developed for assessment of diet in Mediterranean populations [MEDAS (Mediterranean Diet Adherence Screener, MediCul (Mediterranean Diet and Culinary Index), and Mediterranean Oriented Culture Specific FFQ)] [41,42,43]. It contains 14 food groups consisting of 46 food items from the abovementioned FFQs, in addition to products that are commonly consumed in Greece. The FFQ was completed at the first trimester routine antenatal visit of each participant with an oral interview, carried out by trained personnel. Each interview lasted about 20 min.

Adherence to MD was calculated using two scores, the MDS derived by Trichopoulou et al. [28], and the modified MDS derived by Leighton et al. [44]. The latter was used to accommodate lifestyle changes seen in the past years, and compare potential differences between the two scores on the effect on GDM outcome. The MDS developed by Trichopoulou et al. is population specific and is calculated using a 0–9 scoring system; 0 (minimum) relates to no adherence and 9 (maximum) relates to absolute adherence. This tool categorizes foods in 9 components and includes a ratio of monounsaturated lipids to saturated lipids. Subsequently, this score was modified to include PUFA to the MUFA/SFA ratio, and included fruits separate from nuts [20].

The median was calculated for controls, and the value of 1 was assigned for those who had equal or above the mean of the consumption distribution for typical Mediterranean foods (e.g., legumes, fruit and nuts, vegetables, fish, and seafood), whereas the value of 0 was assigned if they had less than the median. On the contrary, for non-Mediterranean foods, including dairy, meat, and meat products, 1 point was awarded when consumption was lower than the median, whereas consumption higher than the median assigned 0 points. For further analysis, the scores from the sampled population achieved were divided in tertiles with the following ranges: 0–3 for low adherence, 4 for middle adherence, and 5–9 for high adherence [28]. The modified MDS by Leighton et al. [44] is a scoring system developed to assess MD adherence and has 14 scoring items. We chose to additionally calculate the adherence to MD with this modified version of MDS score as it is more representative of a Westernized food diet, which nowadays is becoming increasingly popular in Greece. For this modified version, scoring was dependent on the daily or weekly consumption and the values of 0, ½, or 1 were assigned. Food items were grouped in 14 categories. The MDS ranged from 0 (minimum adherence to MD pattern) to 14 (maximum adherence to MD pattern). In order to quantify the scoring system in our population and compare it with the tertiles achieved by the MDS score, population tertiles were again derived: 0–3.5 for low adherence, 4–4.5 for middle adherence, and 5–14 for high adherence. For each component of the MD scoring system, we matched the relevant answers of the FFQ we used. In the absence of an exact match for a food item, the most appropriate for comparison was chosen. For instance, avocado was replaced with olives, whereas for consumption of wine we scored 0, as we did not ask for wine consumption separately, but rather as a general question of alcohol intake.

### 2.4. Statistical Analysis

Comparison of the characteristics between GDM and non-GDM: Continuous data were checked for normality using the Shapiro–Wilk test and P–P plots. Mean (sd) was used to present those normally distributed and median (range) for those skewed. If normally distributed, the student t-test was utilized, and if skewed, the Mann Whitney test was used. Categorical data were presented as relative frequencies and chi-square test was used to assess distribution differences.

Food group comparison between GDM and non-GDM: Since these data were continuous variables, they were checked for normality using the Shapiro–Wilk test. Mean (sd) was used to present those normally distributed and median (range) for those skewed. If normally distributed, the student t-test was utilized, or else the Mann Whitney test was used.

Mediterranean score comparison between GDM and non-GDM: The Mediterranean score was separated into three categories using the population tertiles. These three categories were utilized together with additional characteristics (for computing adjusted OR), i.e., maternal age, BMI, smoking, gravidity, parity, and physical activity, in a multiple logistic regression model. The target binary variable of the model is the GDM outcome. From this model, adjusted odds ratios (aOR) and their 95% confidence intervals (CI) were computed for the Mediterranean score categories.

**Implementation**: The programing language R was used for the implementation.

## 3. Results

A total of 743 pregnant women (age: 32.1 ± 4.85 years) were recruited, of which 112 (15.1%) subsequently developed GDM. Maternal characteristics for the two groups are shown in Table 1. The mean maternal age (33.7 ± 4.9 vs. 31.9 ± 4.9 years, respectively, *p* = 0.0002), as well as the proportion of women aged >35 years old (40.2% vs. 25.7%, *p* = 0.003), were significantly higher in the GDM group. Women that smoked during pregnancy had a higher occurrence of GDM. The mean pre-pregnancy weight and BMI (25.8 ± 5.93 vs. 24.0 ± 4.55, *p* = 0.006) were higher in the GDM group. Additionally, a larger proportion of women with GDM were obese (21.4% vs. 10.6%, *p* = 0.003).

**Figure 1:**
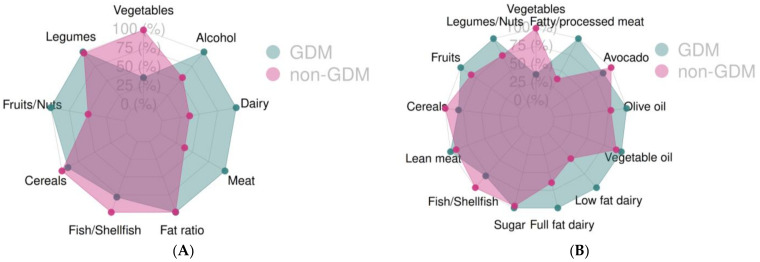
Food group adherence for the two groups, GDM and no GDM, using the Mediterranean Diet Score (**A**) and the modified Mediterranean Diet Score (**B**), GDM: Gestational Diabetes Mellitus.

The food groups for each of the scores used were also compared (Table 2 and Table 3). We observed statistically significant differences for the “meat and derivatives” food group from the MDS index by Trichopoulou et al. (*p* = 0.008) as shown in Table 2. When the modified version of the MDS index by Leighton et al. was used, a significant difference in the “fatty meat and processed meat” food group (Table 3) was observed, with consumption being higher in both cases in the GDM group. No other significant differences were found for the any of the remaining food groups in either score. These differences can be also seen in Figure 1A,B.

**Figure 2:**
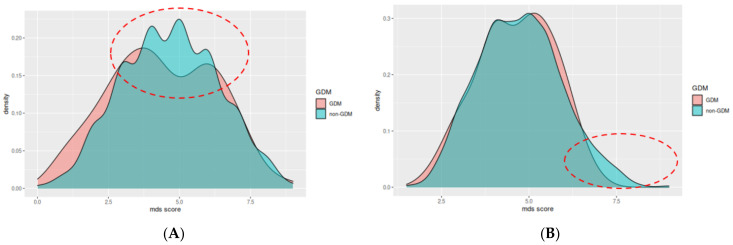
Mediterranean diet index score (**A**) and modified Mediterranean Diet Score (**B**) between the two groups, GDM: Gestational Diabetes Mellitus; MDS score: Mediterranean Diet Score. The main differences are highlighted in the red dotted ellipsoids, were the adherence of the non-GDM group is higher compared to the GDM group.

A score of 5–9 points using the MDS index is associated with 43% lower likelihood of GDM (aOR: 0.57 [95% CI: 0.32, 0.9], *p* = 0.02) (Table 4). Using the modified version of MDS index, we did not observe statistically significant results for any level of adherence (Table 5). Additionally, the overall adherence for each group is shown in Figure 2A,B, where the differences in the adherence scores between the two groups are highlighted in red dotted ellipsoids.

The MD adherence adjusted analysis showed that women who achieved a higher MDS and were in the third tertile (score 5–9) had a 43% lower likelihood of GDM compared to the first tertile (score 0–3, reference level) (aOR: 0.57 95% CI (0.36, 0.94), *p* = 0.02). Results of the analysis can be seen in Table 4. No significant effects were found with the modified MDS score when running the same model by tertile.

## 4. Discussions

To our knowledge, this is the first study that explored the pre-pregnancy MD adherence in relation to GDM occurrence among Greek pregnant women. The main findings were that: 1. high adherence to the original MD diet in the preconception period decreased the risk of GDM, irrespective of pre-pregnancy weight status and other known risk factors, whereas the modified MD had no effect and, 2. pre-pregnancy consumption of “meat and derivatives” and “fatty meat and processed meat” was associated with a higher risk of GDM, with both scoring systems.

Numerous dietary patterns have been associated with various health benefits across the world. The largest data documentation and research though, regard the dietary habits and lifestyle characteristics of populations in the Mediterranean regions [45]. Adherence to the MD has been associated with numerous health benefits for various medical conditions, such as inflammatory bowel disease (IBD) disease, non-alcoholic fatty liver disease (NAFLD), metabolic syndrome, cancer, and longevity [46,47,48,49]. Pregnancies complicated with GDM are associated with an increased risk for developing metabolic syndrome later in life [8]. A recent systematic review and meta-analysis in the non-pregnant population highlighted that adherence to the MD positively affects all parameters of the metabolic syndrome, although further research is needed to specify whether this effect is applicable among healthy and unhealthy individuals [50]. The protective effect of a MD dietary pattern on pregnancy outcomes has also been demonstrated [51].

High level of adherence to the MD before pregnancy was associated with lower incidents of GDM in this study. Previous studies report on the association of MD diet during gestation; a study conducted by Karamanos et al. included participants from 10 Mediterranean countries, including Greek participants. Individuals that had a higher Mediterranean Diet Index (MDI) score were associated with lower incidence of GDM, and moreover, controls with high MDI had a better degree of glucose tolerance [52]. A mother and child cohort, known as the Rhea study [53], which is the largest pregnant cohort in Southern Europe so far, aims to evaluate the effect of many different variables on maternal and childhood outcomes. In particular, the study collects data on nutritional aspects, obesity, neurodevelopment, and progression to asthma in children, as well as environmental and socioeconomic factors. Results of the study demonstrated that higher adherence to the MD during pregnancy was correlated, among other health benefits, with lower childhood adiposity. Moreover, environmental exposure of the mothers to organochlorine pesticides was associated with increased risk for GDM. More studies are needed to address the impact of the many possible causal factors for GDM occurrence, but dietary behavior and lifestyle is clearly underlined. Additionally, how pre-pregnancy behavioral factors are altered or not during the gestational period are areas that need further information, and are part of our ongoing cohort study. Our study collected data of the maternal characteristics and the nutritional preferences among pregnant individuals during the pre-conceptional and gestational period. 

High consumption of “meat and derivatives” and “fatty and processed meat” prior to conception was associated with a higher incidence of GDM, as per our study findings. This comes in accordance with findings from other studies conducted in the pre-conception period [54,55]. A study by Sanchis et al. reported that high total meat intake, particularly red meat, was significantly associated with increased risk for GDM, while no statistical significance was identified for non-heme and total iron intake. Additionally, results from a meta-analysis reported an increased risk of GDM occurrence in individuals that consumed high levels of red and processed meat, saturated fat, and increased cholesterol intake either before or during pregnancy. Furthermore, a study conducted by Liang et al. studied the role of meat and dairy consumption a year prior to conception and also during pregnancy [56]. The study found statistical significance for GDM occurrence when the women had higher intake of total and animal protein in mid gestation. Moreover, high consumption of animal protein may impose a reduced glucose threshold for insulin secretion from the pancreatic β- cells, which will eventually lead to insulin resistance and may subsequently cause inability of the islet cells to produce enough amounts of insulin, leading to the pathogenesis of diabetes [57]. A recent systematic review reported that high consumption of saturated and trans-fat before conception and also during conception can significantly alter glucose homeostasis and pave the way for GDM development [58].

Considering the various effects that maternal pre-conception nutritional and total body fat status may impose on the glucose regulation, combined with the adding effect of the hormonal dysregulations that occur during pregnancy on the glucose homeostasis, the significance of preconception counseling on the nutritional habits of women of childbearing age should be adopted. There is abundant evidence on nutritional guideline recommendations for women during the gestational period. A recent comparative review concluded that most of these standards are in agreement, although some discrepancies still exist [59]. The period before conception should also be given attention, as many nutritional imbalances may exist not only during but also before conception as it is nowadays common of women of reproductive age to be in an over or under nutritional status and have nutritional deficits [60]. Consequently, to attain better outcomes for the pregnancy course, especially in individuals with risk factors for developing GDM, such as advanced maternal age and BMI status, obstetric history of previous GDM pregnancy, family history of GDM, or another situation that increases the risk for GDM incidence, an effective counseling approach, including nutritional behavior, should be followed in order to minimize the risk of developing GDM.

Regarding the strengths in our study, we used two scoring systems, one calculated in two separate ways, and we estimated the score by applying the traditional MDS index, as well as the Westernized version of it. The criteria for the diagnosis of GDM were unanimous, and all participants attended the same antenatal care clinic. We additionally used a culture specific semi-quantative FFQ tailored to fit the Greek dietary habits which was previously validated for use in pregnant women [40].

The study also has certain limitations. The main is the possible recall bias as women were asked to report their diet habits prior to conception. Second, for questions that we were not able to exactly match to a food group to calculate the MDS indices, we either chose a similar one, or none if the latter was not. This may have affected our results towards achieving a lower MD score. Moreover, further subgroup analyses by pre-pregnancy weight status were not performed due to the limited sample size for such analyses. 

To conclude, in a Mediterranean population, higher adherence to the original MD appears to have a protective effect on the occurrence of GDM. Considering that MD is an affordable option to be adapted by low-, middle-, and high-income countries, the need for strategies towards a MD diet and recession from the Westernized dietary patterns is essential. Additionally, the benefits of adapting a MD diet pattern should also be considered by clinicians as a potential hazard reduction tool across women of reproductive age. More research is needed to analyze the impact of other lifestyle components related to the MD populations. 

## Figures and Tables

**Table 1:** Characteristics of individuals with GDM and no GDM.

Maternal Characteristics		Total Population	GDM (*n* = 112, 15.1%)	Non GDM (*n* = 631, 84.9%)	*p* Value
Maternal age in years, mean ± sd		32.1 ± 4.85	33.7 ± 4.54	31.9 ± 4.85	<0.001
Maternal age > 35 years, *n (%)*		207 (27.9)	45 (40.2)	162 (25.7)	0.003
Smoking before pregnancy, *n (%)*		272 (36.6)	40 (35.7)	232 (36.8)	0.92
Never smoked, *n (%)*		397 (53.4)	52 (46.4)	345 (54.7)	0.12
Smoking during pregnancy, *n (%)*		74 (10)	20 (17.9)	54 (8.6)	0.05
Body mass index (kg/m^2^), mean ± sd		24.3 ± 4.82	25.8 ± 5.93	24 ± 4.55	0.006
Weight status pre-pregnancy, *n (%)*	Underweight	29 (3.9)	2 (1.8)	27 (4.3)	0.29
	Normal weight	469 (63.1)	66 (58.9)	403 (63.9)	0.34
	Overweight	154 (20.7)	20 (17.9)	134 (21.2)	0.45
	Obese	91 (12.3)	24 (21.4)	67 (10.6)	0.003
Gravidity, *n (%)*	0	303 (40.8)	38 (33.9)	265 (42)	0.12
	1	256 (34.4)	50 (44.6)	206 (32.7)	0.02
	2	116 (15.6)	14 (12.5)	102 (16.2)	0.4
	3	49 (6.6)	7 (6.3)	42 (6.7)	1
	4	16 (2.1)	3 (2.7)	13 (2.1)	0.72
	5	3 (0.4)	0 (0)	3 (0.5)	1
Parity, *n (%)*	0	395 (53.2)	59 (52.7)	336 (53.3)	0.91
	1	266 (35.8)	43 (38.4)	223 (35.3)	0.6
	2	72 (9.7)	9 (8)	63 (10)	0.6
	3	10 (1.4)	1 (0.9)	9 (1.5)	1

Body mass index (BMI) classifications, based on pre-pregnancy weight: underweight BMI < 18.5 kg/m^2^, normal weight BMI 18.5–24.9 kg/m^2^, overweight BMI 25–29.9 kg/m^2^, and obese BMI > 30 kg/m^2^. Between group differences were tested using Student *t*-test for continuous variables and chi square test for categorical. Significance level (alpha) set at 5%.

**Table 2:** Food group categories of the Mediterranean diet and mean consumption in the two groups, GDM and non GDM, based on the MDS.

Food Group Categories of the Mediterranean Diet (g/Day)	GDM (*n* = 112) Median 95% CI	Non GDM (*n* = 631) Median 95% CI	*p*-Value
	p25, p50, p75	p25, p50, p75	
Vegetables	88.3 83.3, 88.3 60.3, 88.3, 117.9	92.7 88.3–98.8 63, 92.7, 133.3	0.08
Legumes	26.7 26.7–26.7 26.78, 26.7, 26.7	26.7 26.7–26.7 26.78, 26.7, 26.7	0.86
Fruits and nuts	130 92.8–134.5 51.3, 130, 175.2	119.5 94.3–130 55.7, 119.5, 155.8	0.86
Cereals	12.1 6.09–18.2 0, 12.1, 24.3	12.1 12.1–12.1 0, 12.1, 18.2	0.93
Fish	18.2 12.7–18.2 8.5, 18.2, 18.2	18.2 18.2–18.2 8.5, 18.2, 18.2	0.34
Dairy products	182.7 120.8–246.1 120.8, 182.7, 285.5	164.7 144.3–167.8 120.8, 164.7, 241.6	0.24
Meat and derivatives	52.1 36–56 36, 52.1, 73.1	41.4 36–46.1 36, 41.4, 56	0.008
Monounsaturated/saturated fat ratio	1.53 1.4–1.7 1.2, 1.5, 1.9	1.53 1.48–1.58 1.28, 1.53, 1.8	0.86
Alcohol	0 0–11 0, 0, 29.5	0 0–0 0, 0, 23.6	0.11
MDS score, mean (sd)	4.4 (1.8)	4.7 (1.7)	0.18

MDS: Mediterranean Diet Score; CI: confidence interval; g/day: grams/day; ND: no data. Mediterranean diet adherence assessed using the MDS index by Trichopoulou et al. [28]. Method for statistical analysis: Mann Whitney test since data not normally distributed.

**Table 3:** Food group categories of the Mediterranean diet and mean consumption in the two groups, GDM and non GDM, using the modified MDS index.

Food Group Categories of the Mediterranean Diet (g/Day)	GDM (*n* = 112) Median 95% CI	Non GDM (*n* = 631) Median 95% CI	*p* Value
	p25, p50, p75	p25, p50, p75	
Vegetables (without potatoes)	88.3 83.3–88.3 60.3, 88.3, 117.9	92.7 88.3–98.8 63, 92.7, 133.3	0.07
Legumes and nuts	33.2 26.7–45.2 26.7, 33.2, 56.9	33.2 31.3–39.7 26.7, 33.2, 52.6	0.46
Fruits	132 115.4–174.5 53.5, 132, 255	130 130–134.1 64.2, 130, 201.4	0.95
Whole grain cereals	16.7 12.1–18.2 0, 16.7, 24.3	12.1 12.1–18.2 1.4, 12.1, 24.3	0.89
Lean meat	16 16–16 16.07, 16.07, 32.14	16 16–16 16, 16, 32.1	0.67
Fish and shellfish	18.2 12.7–18.2 8.5, 18.21, 18.21	18.2 18.2–18.2 8.5, 18.21, 18.21	0.34
Fatty meat and processed meat	47 40–58.4 31.9, 47.04, 72.2	40 38.4–40 22.8, 40, 60	0.004
Full fat dairy products not fermented	120.8 120.8–120.8 34.5, 120.8, 241.6	120.8 120.8–120.8 51.77, 120.8, 241.6	0.72
Low fat and fermented dairy products	0 0–0 0, 0, 123.56	0 0–0 0, 0, 70.60	0.78
Vegetable oils	0 0–0 0, 0, 0	0 0–0 0, 0, 0	0.33
Olive and canola oil	30 30–30 15, 30, 30	30 30–30 15, 30, 45	0.32
Avocado	1.3 0–2.6 0, 1.33, 5.71	2.6 1.3–2.6 0, 2.6, 11.4	0.40
Sugar	19.8 13.2–26.4 4.6, 19.8, 46.3	19.8 19.8–19.8 6.6, 19.8, 46.3	0.86
Wine	ND	ND	ND
Modified MDS, mean (sd)	4.6 (1.1)	4.7 (1.2)	0.62

CI: confidence interval; g/day: grams/day; ND: no data; Mediterranean diet adherence assessed using the modified MDS index by Leighton et al. [44]. Method for statistical analysis: Mann Whitney since data did not follow the normal distribution.

**Table 4:** Level of adherence to MDS, in total and by GDM status and likelihood of GDM by level of MDS adherence, using multiple logistic regression analysis.

Adherence to Mediterranean Diet (Points)	Total Population *n* (%)	GDM *n* (%)	Non GDM *n* (%)	aOR	95% CI	*p* Value
Low (0–3)	202 (27.2)	38 (33.9)	164 (26)	1	Reference	Reference
Middle (4)	152 (20.5)	23 (20.5)	129 (20.44)	0.72	0.40–1.28	0.27
High (5–9)	389 (52.4)	51 (45.5)	338 (53.6)	0.57	0.36–0.94	0.02

Model adjusted for maternal age, BMI, smoking, gravidity, parity, physical activity using the MDS scoring scale by Trichopoulou et al. [28]; Significance level set at 5%. GDM: Gestational Diabetes Mellitus; aOR: adjusted odds ratio.

**Table 5:** Level of adherence to modified MDS, in total and by GDM status and likelihood of GDM by level of modified MDS adherence, using multiple logistic regression analysis. Modified MDS.

Adherence to Mediterranean Diet (Points)	Total Population *n* (%)	GDM (%)	Non GDM *n* (%)	aOR	95% CI	*p* Value
Low (0–3.5)	157 (21.13)	23 (20.54)	134 (21.23)	1	Reference	Reference
Middle (4–4.5)	341 (45.9)	53 (47.32)	288 (45.64)	1.02	0.59–1.78	0.94
High (5–14)	245 (32.97)	36 (32.14)	209 (33.12)	0.85	0.47–1.55	0.59

GDM: Gestational Diabetes Mellitus; aoR: adjusted odds ratio; Model adjusted for maternal age, BMI, smoking, gravidity, parity, and physical activity using the scoring scale by Leighton et al. [44]; Level of adherence was calculated by in two categories, distributing the data for low and high adherence. Significance level set at 5%.

## Data Availability

The data presented in this study are not publicly available due to privacy restrictions.
